# Spinal intradural hematoma after intrathecal drug delivery system implantation in a patient with possible heparin-induced thrombocytopenia: a case report

**DOI:** 10.3389/fonc.2026.1880749

**Published:** 2026-08-04

**Authors:** Zhengqiong Cheng, Aimin Zhang

**Affiliations:** 1 Department of Anesthesiology, Sichuan Gem Flower Hospital, Chengdu, China; 2 Department of Anesthesiology, Sichuan Clinical Research Center for Cancer, Sichuan Cancer Hospital & Institute, Sichuan Cancer Center, University of Electronic Science and Technology of China, Chengdu, China

**Keywords:** cancer pain, case report, enoxaparin, heparin-induced thrombocytopenia, intrathecal drug delivery system, spinal intradural hematoma

## Abstract

**Background:**

Intrathecal drug delivery systems (IDDSs) can provide analgesia for selected patients with refractory cancer pain. Neuraxial implantation in patients receiving antithrombotic therapy carries a risk of spinal bleeding. Heparin-induced thrombocytopenia (HIT) is mainly prothrombotic and should be diagnosed by combining clinical probability with laboratory testing.

**Case presentation:**

A 50-year-old woman with metastatic rectal adenocarcinoma, refractory cancer pain, previous lower-extremity venous thromboembolism, and chronic aspirin use underwent image-guided IDDS implantation after peri-procedural enoxaparin exposure. Enoxaparin 1.5 mg/kg was stopped 24 hours before implantation. On the same day, she developed acute left lower-extremity weakness (2/5) and blood-tinged cerebrospinal fluid from the intrathecal catheter. Platelets fell from 145 × 10^9^/L to 40 × 10^9^/L, and hemoglobin was 54 g/L. MRI showed a T12–S2 spinal intradural hematoma compressing the conus medullaris and cauda equina. A heparin-induced platelet activation assay on postoperative day 2 was reported as strongly positive. Management included transfusion support, corticosteroids, neurotrophic support, lumbar cerebrospinal fluid drainage, and hematological stabilization. Strength improved to 4/5 by postoperative day 6. Intrathecal analgesia was resumed on postoperative day 12, and the pain score decreased to 3/10.

**Conclusion:**

This case describes spinal intradural hematoma with acute thrombocytopenia after peri-procedural enoxaparin exposure. The findings support possible HIT but do not show that HIT directly caused the hematoma. The event was likely multifactorial. Acute platelet decline after heparin exposure and new neurological deficits after a neuraxial procedure require prompt evaluation and multidisciplinary management.

## Introduction

Intrathecal drug delivery is used in selected patients with refractory cancer pain when systemic therapy provides inadequate analgesia or unacceptable toxicity. Current Polyanalgesic Consensus Conference guidance emphasizes individualized patient selection, assessment of comorbidities, technical safety, medication management, and multidisciplinary decision-making (1).

Neuraxial procedures in patients receiving antithrombotic therapy require careful review of the treatment indication, drug-specific interruption interval, renal function, bleeding risk, and the consequences of neurological compression. Current ASRA, ESAIC/ESRA, and interventional pain guidelines address these issues because spinal canal bleeding, although rare, may cause permanent neurological injury (2–4).

We report a spinal intradural hematoma after IDDS implantation in a patient with advanced cancer, peri-procedural enoxaparin exposure, acute thrombocytopenia, and a strongly positive heparin-induced platelet activation (HIPA) assay. The event is interpreted as possible HIT with multifactorial spinal bleeding, rather than proof that HIT directly caused the hematoma.

A 50-year-old woman with stage IV rectal adenocarcinoma and multifocal metastatic disease presented with refractory cancer-related pain rated 8–9/10 on the Numerical Rating Scale (NRS) despite high-dose oral opioid therapy. Her medical history included lower-extremity venous thromboembolism and chronic aspirin use at 100 mg/day.

After shared decision-making with the patient and her family, the multidisciplinary team selected IDDS implantation to improve pain control and quality of life. Aspirin was stopped after admission, and subcutaneous enoxaparin 1.5 mg/kg was started. The last dose was given 24 hours before the procedure. Preprocedural testing showed a D-dimer level of 34.51 µg/mL, an international normalized ratio of 1.36, and a platelet count of 145 × 10^9^/L.

Image-guided IDDS implantation was performed on June 25, 2025, and was described as uneventful.

Following implantation on the same calendar day, the patient developed acute left lower-extremity weakness with Medical Research Council strength of 2/5. Blood-tinged cerebrospinal fluid was aspirated through the intrathecal catheter, and the intrathecal infusion was stopped immediately. Repeat testing showed a platelet count of 40 × 10^9^/L, representing a decrease of more than 50% from the preprocedural value, and a hemoglobin level of 54 g/L.

**Figure 1:**
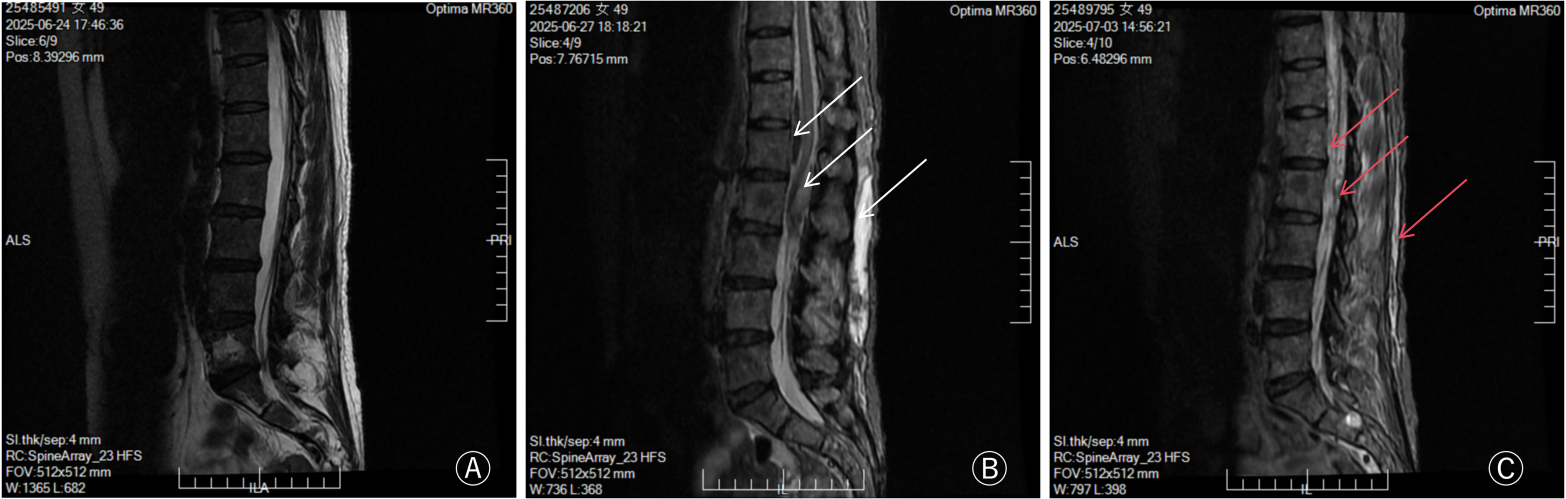
Serial T2-weighted MRI of the lumbar spine. **(A)** Preoperative sagittal MRI showed posterior disc bulging at L3–L4 and L4–L5. **(B)** MRI obtained after neurological deterioration showed mixed-signal material within the spinal canal from T12 to S2, with compression and edema of the conus medullaris and cauda equina. The MRI appearance did not clearly distinguish among subarachnoid, subdural, or mixed intradural compartments; the lesion is therefore described as a spinal intradural hematoma. **(C)** Follow-up MRI obtained after left lower-extremity strength improved to 4/5 showed residual streak-like intradural signal abnormalities anterior to the conus medullaris at T12–L1, posterior to the cauda equina at L2–L3, and at L5–S2, with reduced hematoma extent and less compression than on the prior study.

Spinal MRI showed an extensive T12–S2 intradural hematoma with compression of the conus medullaris and cauda equina (**Figure 1**). The MRI appearance did not clearly distinguish among subarachnoid, subdural, or mixed intradural compartments. The term “spinal intradural hematoma” is therefore used throughout this report.

Conservative management included red-cell, plasma, and platelet transfusion, high-dose corticosteroids, neurotrophic support, lumbar cerebrospinal fluid drainage, and hematological stabilization. Neurosurgical decompression was not performed.

On postoperative day 2, a HIPA assay was reported as strongly positive. Together with the platelet decrease of more than 50% after enoxaparin exposure, this result raised concern for HIT. Because HIT is a clinicopathologic diagnosis, the case is described as possible HIT rather than definite HIT.

By postoperative day 6, left lower-extremity strength had improved to 4/5. Intrathecal analgesia was resumed on postoperative day 12, and the NRS pain score decreased to 3/10.

## Discussion

This case illustrates the difficulty of balancing analgesic benefit, thromboembolic risk, and neuraxial bleeding risk in a patient with advanced cancer. Current PACC recommendations support individualized selection for intrathecal therapy and careful assessment of comorbidities, procedural feasibility, medication safety, prognosis, and patient goals (1). Palliative intent does not reduce the need for standard procedural safeguards.

Advanced cancer can increase both thrombotic and bleeding risk through complex changes in hemostasis (10). In this patient, the elevated D-dimer and mildly prolonged international normalized ratio indicated abnormal hemostasis but did not establish disseminated intravascular coagulation. Severe thrombocytopenia and profound anemia may also have increased the risk of bleeding after neuraxial instrumentation.

The platelet decrease of more than 50% after enoxaparin exposure and the strongly positive HIPA assay raised concern for HIT. However, HIT is a clinicopathologic diagnosis that requires assessment of clinical probability together with laboratory findings (6–8). A positive functional assay supports the diagnosis but does not by itself establish that HIT caused the hematoma. The most appropriate description in this case is therefore possible HIT.

HIT is an immune-mediated, platelet-activating, and predominantly prothrombotic disorder (6, 7). Low-molecular-weight heparin has a lower incidence of HIT than unfractionated heparin, but the risk is not zero (9). Major bleeding is not a typical direct manifestation of HIT. In this case, severe thrombocytopenia, possible neuraxial vessel injury, cancer-related hemostatic abnormalities, and anemia may have acted together to lower the threshold for spinal bleeding. The findings show a temporal association rather than a single causal pathway.

The perioperative antithrombotic strategy also requires careful review. Aspirin is not standard treatment for active or recent venous thromboembolism, and interruption of aspirin is not usually bridged in the same way as interruption of a vitamin K antagonist. Current guidance reserves bridging anticoagulation for selected patients receiving long-term anticoagulation who have a sufficiently high thromboembolic risk (5). The documented 24-hour interval between the last 1.5 mg/kg enoxaparin dose and neuraxial implantation was consistent with the minimum interval recommended for high-dose low-molecular-weight heparin (2–4), but this interval does not eliminate the risks of bleeding or HIT.

New neurological weakness after a neuraxial procedure requires urgent assessment for spinal canal bleeding. MRI is the key diagnostic study, and management should be individualized according to neurological severity, hematoma location and progression, coagulation status, and surgical feasibility (11, 12). This patient improved during conservative management, but a single case cannot determine whether conservative treatment or surgical decompression is preferable.

When HIT is suspected, current guidance supports assessment of clinical probability, discontinuation of heparin in patients with intermediate or high probability, appropriate laboratory testing, and consideration of non-heparin anticoagulation while accounting for active bleeding risk (6, 7). A single case cannot support a universal platelet-monitoring protocol. The practical message is that an otherwise unexplained platelet decrease of more than 50% after heparin exposure should prompt urgent HIT evaluation, and any new neurological deficit after a neuraxial procedure should prompt spinal imaging and neurosurgical review.

## Limitations and avenues for future investigation

It highlights a significant knowledge gap: robust, evidence-based protocols for perioperative anticoagulation management specifically tailored to cancer patients, particularly those with advanced disease and complex coagulopathies, are lacking. Prospective studies are urgently needed to:

• Quantify the incidence of HIT and other heparin-related complications in cancer patients undergoing bridging therapy for neuraxial procedures.
• Develop and validate risk-stratification models that incorporate cancer type, coagulation profile, metastatic burden, and type/duration of heparin exposure.
• Evaluate the efficacy and cost-effectiveness of routine platelet monitoring during bridging therapy in this high-risk cohort.
• Explore alternative bridging strategies or non-neuraxial analgesic options for patients deemed at exceptionally high risk.

This report has several limitations. The available records did not contain a complete antithrombotic history or enoxaparin dosing schedule, detailed procedural variables, serial platelet and hemoglobin measurements, baseline hemoglobin and hemolysis results, sufficient information for a reliable 4Ts score, full HIPA assay methodology, or a comprehensive assessment of alternative causes of thrombocytopenia and anemia. The exact intradural compartment, rationale for each treatment decision, and detailed neurological follow-up were also not available. These limitations restrict assessment of the diagnosis, mechanism, and management. The report therefore identifies a temporal association among enoxaparin exposure, acute thrombocytopenia, and spinal intradural hematoma, but it does not establish causation. The case is reported in accordance with the CARE principles as far as the available information permits (13).

## Conclusion

It advocates for a paradigm of extreme caution, characterized by:

• Pre-operative Multidisciplinary Consensus: Involving pain specialists, palliative care physicians, hematologists, and oncologists to perform a holistic risk-benefit analysis that includes specific HIT risk assessment.
• Enhanced Vigilance: Considering preoperative platelet count monitoring during bridging therapy to detect early trends suggestive of HIT.
• Explicit, Informed Consent: Ensuring patients and families understand not only the general risk of spinal hematoma but also the specific, added risk profile associated with heparin bridging, including HIT.
• Palliative Care Integration: Anchoring all decisions in patient-defined goals of care, ensuring that the pursuit of pain relief remains consonant with the overarching objectives of quality of life and symptom control.
• The laboratory confirmation of HIT via functional assay in this case reinforces the need for such a cautious and comprehensive approach. In the delicate domain of palliative oncology, where the intent is to alleviate suffering, our interventions must be as sophisticated and carefully calibrated as the pathologies we treat. Further research is imperative to refine our protocols and safeguard this vulnerable population during their most challenging times.

Acute platelet decline after peri-procedural heparin exposure should prompt evaluation for HIT and other causes of thrombocytopenia. New neurological deficits after IDDS implantation require urgent assessment for spinal bleeding. This case suggests a multifactorial event involving antithrombotic exposure, severe thrombocytopenia, possible procedural injury, cancer-related hemostatic abnormalities, and anemia. Management should follow established HIT, antithrombotic, neuraxial-procedure, and intrathecal-therapy guidance and should involve multidisciplinary review.

## Data Availability

The original contributions presented in the study are included in the article/supplementary material. Further inquiries can be directed to the corresponding author.

## References

[1] Deer TR Hayek SM Grider JS Pope JE Brogan SE Gulati A . The polyanalgesic consensus conference (PACC)®: Updates on clinical pharmacology and comorbidity management in intrathecal drug delivery for cancer pain. Neuromodulation. (2025) 28:1029–53. doi: 10.1016/j.neurom.2024.08.006 39297833

[2] Kopp SL Vandermeulen E McBane RD Perlas A Leffert L Horlocker T . Regional anesthesia in the patient receiving antithrombotic or thrombolytic therapy: American Society of Regional Anesthesia and Pain Medicine evidence-based guidelines (Fifth Edition). Reg Anesth Pain Med. (2025):rapm-2024-105766. doi: 10.1136/rapm-2024-105766 39880411

[3] Kietaibl S Ferrandis R Godier A Llau J Lobo C Macfarlane AJR . Regional anaesthesia in patients on antithrombotic drugs: Joint ESAIC/ESRA guidelines. Eur J Anaesthesiol. (2022) 39:100–32. doi: 10.1097/EJA.0000000000001600 34980845

[4] Narouze S Benzon HT Provenzano D Buvanendran A De Andres J Deer T . Interventional spine and pain procedures in patients on antiplatelet and anticoagulant medications (Second Edition): Guidelines from ASRA, ESRA, AAPM, INS, NANS, and WIP. Reg Anesth Pain Med. (2018) 43:225–62. doi: 10.1097/AAP.0000000000000700 29278603

[5] Douketis JD Spyropoulos AC Murad MH Arcelus JI Dager WE Dunn AS . Perioperative management of antithrombotic therapy: An American College of Chest Physicians clinical practice guideline. Chest. (2022) 162:e207–43. doi: 10.1016/j.chest.2022.07.025 35964704

[6] Cuker A Arepally GM Chong BH Cines DB Greinacher A Gruel Y . American Society of Hematology 2018 guidelines for management of venous thromboembolism: Heparin-induced thrombocytopenia. Blood Adv. (2018) 2:3360–92. doi: 10.1182/bloodadvances.2018024489 30482768 PMC6258919

[7] May J Cuker A . Practical guide to the diagnosis and management of heparin-induced thrombocytopenia. Hematol Am Soc Hematol Educ Program. (2024) 2024:388–95. doi: 10.1182/hematology.2024000566 39644042 PMC11665626

[8] Cuker A Gimotty PA Crowther MA Warkentin TE . Predictive value of the 4Ts scoring system for heparin-induced thrombocytopenia: A systematic review and meta-analysis. Blood. (2012) 120:4160–7. doi: 10.1182/blood-2012-07-443051 22990018 PMC3501714

[9] Martel N Lee J Wells PS . Risk for heparin-induced thrombocytopenia with unfractionated and low-molecular-weight heparin thromboprophylaxis: A meta-analysis. Blood. (2005) 106:2710–5. doi: 10.1182/blood-2005-04-1546 15985543

[10] Tsantes AG Petrou E Tsante KA Sokou R Frantzeskaki F Domouchtsidou A . Cancer-associated thrombosis: Pathophysiology, laboratory assessment, and current guidelines. Cancers (Basel). (2024) 16:2082. doi: 10.3390/cancers16112082 38893201 PMC11171168

[11] Bauer ME Toledano RD Houle T Beilin Y MacEachern M McCabe M . Lumbar neuraxial procedures in thrombocytopenic patients across populations: A systematic review and meta-analysis. J Clin Anesth. (2020) 61:109666. doi: 10.1016/j.jclinane.2019.109666 31810860

[12] Kumar N Palmisciano P Dhawan S Boakye M Drazin D Sharma M . Spontaneous spinal hematoma in patients using antiplatelets and anticoagulants: A systematic review. World Neurosurg. (2024) 184:e185–94. doi: 10.1016/j.wneu.2024.01.082 38278210

[13] Riley DS Barber MS Kienle GS Aronson JK von Schoen-Angerer T Tugwell P . CARE guidelines for case reports: Explanation and elaboration document. J Clin Epidemiol. (2017) 89:218–35. doi: 10.1016/j.jclinepi.2017.04.026 28529185

